# Porcine placental extract increase the cellular NAD levels in human epidermal keratinocytes

**DOI:** 10.1038/s41598-022-23446-9

**Published:** 2022-11-09

**Authors:** Takeshi Katayoshi, Nobuaki Yamaura, Takahisa Nakajo, Natsuko Kitajima, Kentaro Tsuji-Naito

**Affiliations:** DHC Corporation Laboratories, Division 2, 2-42 Hamada, Mihama-ku, Chiba, 261-0025 Japan

**Keywords:** Biological techniques, Cell biology, Molecular biology, Health care

## Abstract

Nicotinamide adenine dinucleotide (NAD) is an essential cofactor for numerous enzymes involved in energy metabolism. Because decreasing NAD levels is a common hallmark of the aging process in various tissues and organs, maintaining NAD levels has recently been of interest for the prevention of aging and age-related diseases. Although placental extract (PE) are known to possess several anti-aging effects, the NAD-boosting activity of PE remains unknown. In this study, we found that porcine PE (PPE) significantly increased intracellular NAD levels in normal human epidermal keratinocytes (NHEKs). PPE also attenuated the NAD depletion induced by FK866, an inhibitor of nicotinamide phosphoribosyltransferase (NAMPT). Interestingly, only the fraction containing nicotinamide mononucleotide (NMN), nicotinamide riboside (NR), and nicotinamide (NAM) restored NAD content in NHEKs in the absence of NAMPT activity. These results suggest that PPE increases intracellular NAD by providing NAD precursors such as NMN, NR, and NAM. Finally, we showed that the application of PPE to the stratum corneum of the reconstructed human epidermis significantly ameliorated FK866-induced NAD depletion, suggesting that topical PPE may be helpful for increasing skin NAD levels. This is the first study to report the novel biological activity of PE as an NAD booster in human epidermal cells.

## Introduction

Nicotinamide adenine dinucleotide (NAD) is a classical cofactor for many redox reactions and is present in all living cells in the oxidized (NAD^+^) and reduced (NADH) forms. NAD^+^ is also known as an essential co-substrate for NAD^+^-consuming enzymes such as sirtuin (SIRT) and poly ADP-ribose polymerase (PARP). Because these enzymes play a central role in various biological processes, including DNA repair, mitochondrial metabolism, and stress resistance, imbalanced NAD homeostasis affects their pivotal processes^1^. Tissue/organ NAD levels decline with age, resulting in imbalanced NAD homeostasis^2–6^. Therefore, there is now a consensus that maintaining NAD levels leads to the prevention and treatment of aging and age-related diseases^7^. NAD is biosynthesized via a variety of pathways, including de novo biosynthesis from tryptophan, the Preiss–Handler pathway from nicotinic acid, and the salvage pathway from nicotinamide (NAM)^8^. In mammals, the salvage pathway is the major pathway for maintaining cellular NAD levels^9^. In this pathway, NAM is converted to nicotinamide mononucleotide (NMN) by NAM phosphoribosyltransferase (NAMPT) and is then converted to NAD by NMN adenylyltransferases. Nicotinamide riboside (NR) is converted to NMN by NR kinases and then integrated into the salvage pathway. NAD intermediate metabolites, such as NMN and NR, have been shown to elevate NAD levels in various cells and tissues and improve age-related diseases^10,11^.

Placental extract (PE) has been widely used in traditional medicine for wound healing. PE is a rich source of numerous bioactive components, including amino acids, nucleic acids, fatty acids, minerals, vitamins, hormones, bioactive peptides, cytokines, and growth factors. It is known to have various pharmacological activities, such as anti-inflammatory, anti-oxidant, regenerative, and immunomodulatory effects^12^. Thus, subcutaneous and intramuscular injections of human PE (HPE) have been clinically used for the treatment of several diseases, including menopausal symptoms and chronic liver disease^13^. Furthermore, HPE is used clinically as an anti-wrinkle agent mainly in Asian countries because of its effects on cell growth and collagen synthesis^14^. Since the use of HPE is restricted to medical applications in some countries such as Japan, porcine PE (PPE) was recently developed as an alternative to HPE and used as a raw ingredient in health foods and cosmetics. An in vivo study using a mouse contact dermatitis model showed that topical PPE has anti-inflammatory and antioxidative effects on the skin^15^. Clinical studies have reported that topical PPE is beneficial for the treatment of chronic non-healing wounds and preventing wrinkle formation^16,17^. Furthermore, a randomized, double-blind, placebo-controlled clinical trial was conducted in healthy adult women to validate the skin benefits of oral ingestion of PPE and showed that the skin condition of the PPE intake group was significantly improved^18^. These reports support the concept that PPE treatment can alleviate or prevent skin aging.

PE has been conventionally used as an anti-aging agent, and numerous clinical studies have established its anti-aging properties. However, the mechanism underlying the anti-aging effects of PE remains unclear. This report showed that PPE contains several NAD precursors, such as NMN and NR, and increases intracellular NAD levels in normal human epidermal keratinocytes (NHEKs). Our findings provide new insights into the mechanism through which PE exerts its anti-aging effects by maintaining intracellular NAD homeostasis.

## Results

### PPE increases the intracellular NAD levels

**Figure 1:**
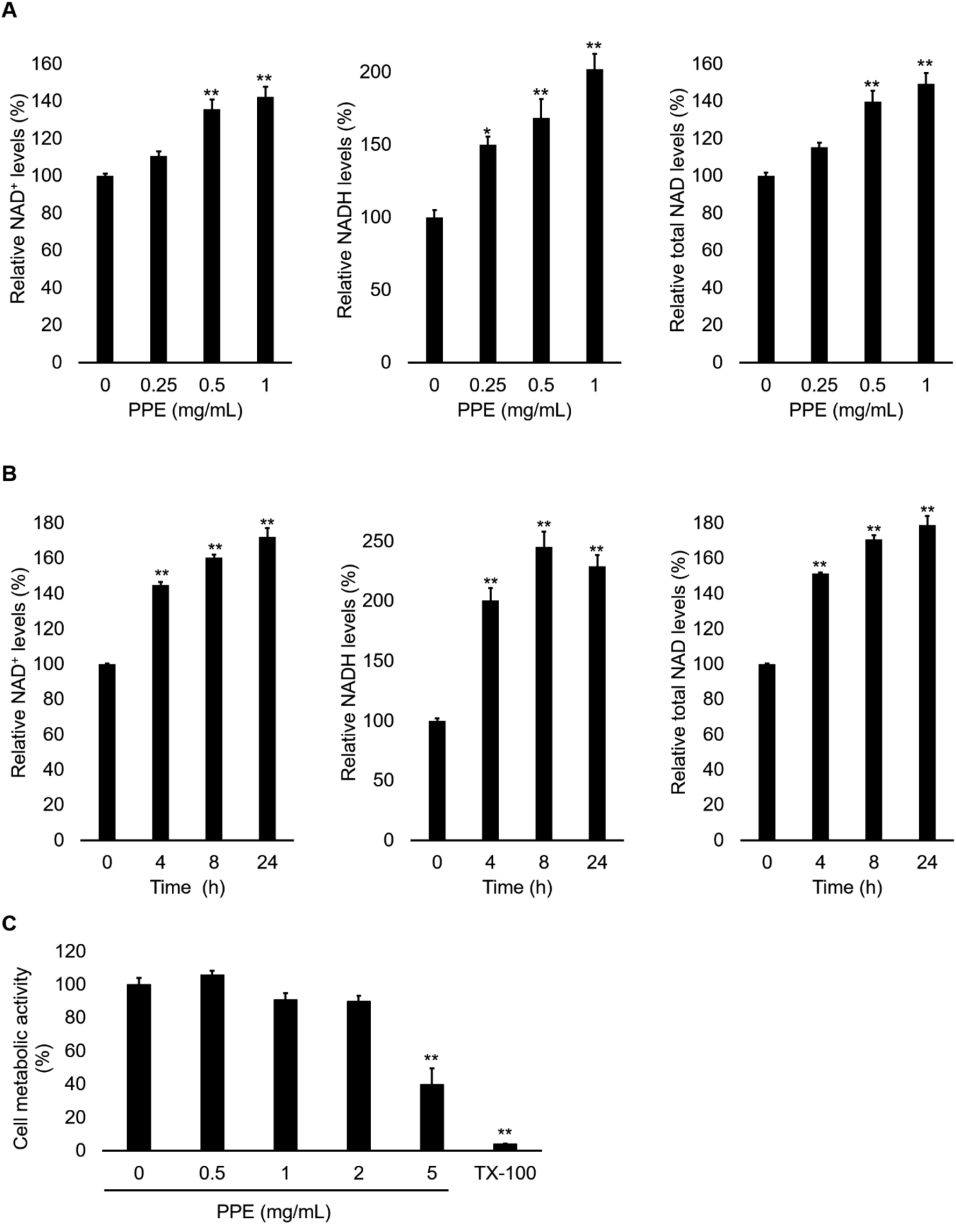
PPE increases cellular NAD levels in NHEKs. The NHEKs were incubated with PPE at the indicated concentration or 0.2% TX-100 (as the positive control) for 24 h (**A,C**) or 1 mg/mL PPE for the indicated time (**B**). The cellular NAD^+^, NADH, and total NAD levels were determined using NAD^+^/NADH assay kit. The cell metabolic activity was determined using the MTT assay. *p < 0.05 and **p < 0.01, in comparison with the control.

To investigate whether PPE changes cellular NAD states, we cultured NHEKs in a medium supplemented with PPE and quantified their intracellular NAD^+^ and NADH levels. The results showed that PPE significantly increased the amount of both NAD^+^ and NADH in NHEKs in a dose-dependent manner (Fig. 1A). The total NAD (NAD^+^  + NADH) level of the cells treated with 1 mg/mL PPE was approximately 1.5 times higher than that of untreated cells (Fig. 1A). In addition, in a time-course experiment, significant elevation of NAD^+^, NADH, and total NAD levels was observed after 4 h of treatment with 1 mg/mL PPE (Fig. 1B). The 3-(4,5-dimethylthiazol-2-yl)-2,5-diphenyltetrazolium bromide (MTT) assay confirmed that 0.5–2 mg/mL PPE had no effect on cell metabolic activity, whereas 5 mg/mL PPE or the positive control (0.2% Triton X-100 [TX-100]) exhibited significant cell toxicity (Fig. 1C). As NAD was hardly detected in PPE itself (data not shown), the NAD-producing effect of PPE did not seem to be due to the influx of PPE-derived NAD into NHEKs. Thus, these results suggest that PPE can increase intracellular NAD levels.

### Active ingredients of PPE are small molecules not proteins

**Figure 2:**
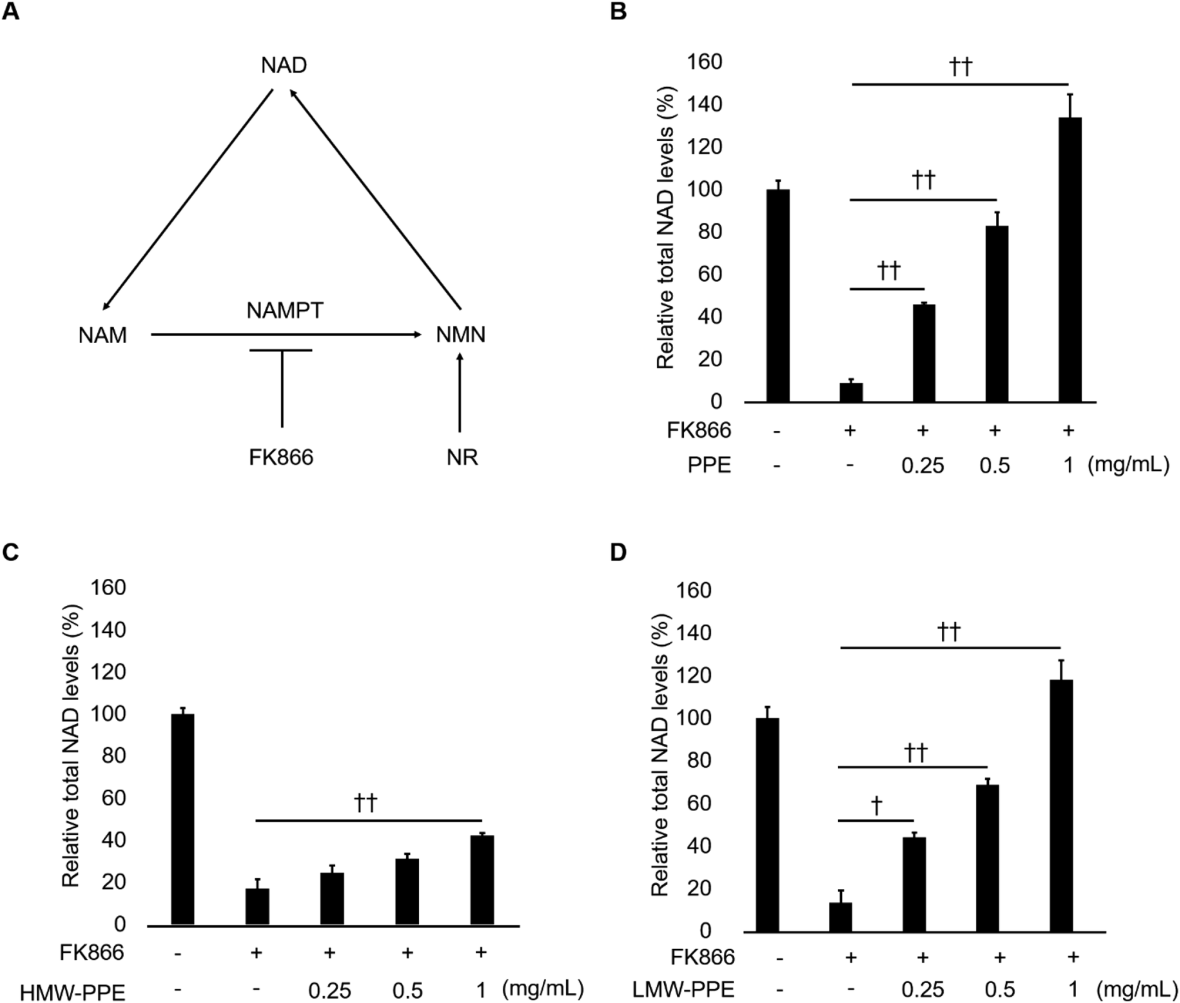
PPE ameliorates the depletion of NAD by FK866 in NHEKs. (**A**) The salvage pathway of NAD biosynthesis. The NHEKs were pre-treated with FK866 for 30 min and incubated with whole-PPE (**B**), HMW-PPE (**C**), or LMW-PPE (**D**) at the indicated concentrations for 24 h. The total NAD levels were determined using NAD^+^/NADH assay kit. ^†^*p* < 0.05 and ^††^*p* < 0.01, in comparison with the indicated groups.

To determine whether PPE boosts cellular NAD biosynthesis via the NAMPT reaction in the NAD salvage pathway, we performed a NAD recovery assay using the NAMPT inhibitor FK866 (Fig. 2A). FK866 considerably reduced the total NAD levels 24 h after addition (Fig. 2B). Interestingly, despite the absence of NAMPT activity within NHEKs, PPE suppressed FK866-induced depletion of NAD in a dose-dependent manner (Fig. 2B). Many reports have shown that PE contains proteins such as cytokines and growth factors^13,19,20^. Therefore, we presumed that some bioactive proteins or porcine NAMPT contained in PPE might enhance intracellular NAD biosynthesis in NHEKs. To confirm our hypothesis, we separated PPE into high- and low-molecular-weight (HMW, LMW) fractions using a centrifugal filter device (cutoff: 3 kDa). Since the NAD recovery assay using FK866 showed the effect of PPE on NAD biosynthesis more clearly than in the absence of FK866 (Figs. 1A and 2B), we proceeded to identify the active ingredients in PPE using this assay. Unexpectedly, HMW-PPE required a high concentration of 1 mg/mL for significant restoration of NAD levels, while the LMW-PPE clearly ameliorated the FK866-induced NAD depletion even at a low concentration of 0.25 mg/mL (Fig. 2C and D). Thus, these results suggest that the primary active ingredients in PPE are small molecules of less than 3 kDa, rather than proteins such as growth factors and cytokines.

### PPE contains NAD precursors, such as NMN and NR

**Figure 3:**
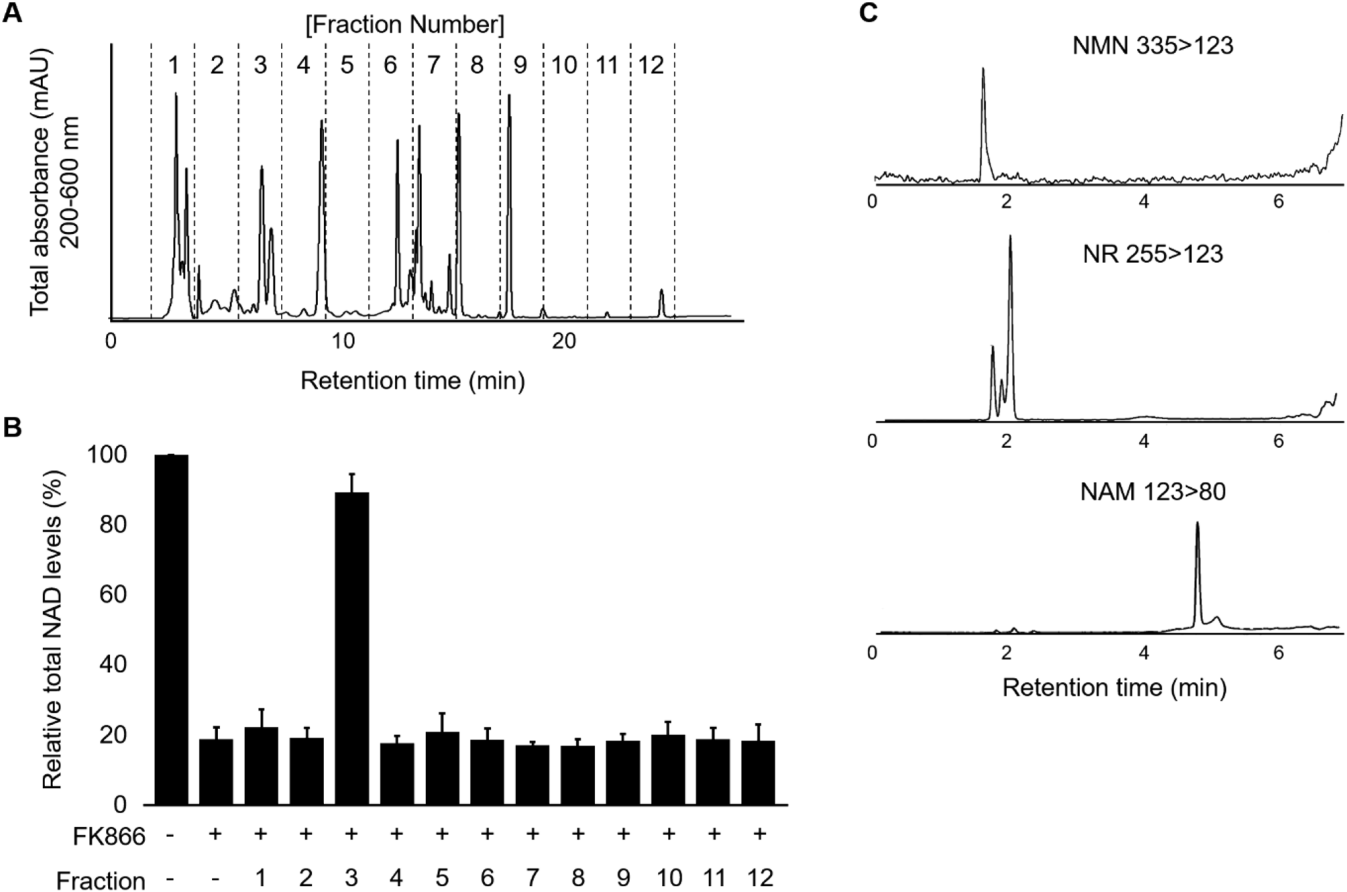
The active fraction of LMW-PPE contains several NAD precursors. (**A**) LMW-PPE (1 mg) was separated and monitored at the multiple-wavelength (200–600 nm) using HPLC. (**B**) The eluents were collected per 2 min and tested for NAD recovery activity in each fraction. (**C**) The fraction 3 was analyzed using LC–MS/MS. All compounds were detected in positive ion mode.

We further fractionated LMW-PPE into 12 fractions using reversed-phase high performance liquid chromatography (HPLC) and investigated which fraction contributed to the restoration of FK866-induced NAD depletion. A typical HPLC chromatogram of the LMW-PPE is shown in Fig. 3A. The NAD recovery assay showed that only fraction 3 distinctly increased NAD levels in FK866-treated NHEKs (Fig. 3B). NAD precursors, such as NMN and NR, elevate intracellular NAD levels without NAMPT intervention in various cell lines including NHEKs^10,21^. Since LMW-PPE still increased NAD content in NHEKs, even when NAMPT was inhibited by FK866, we hypothesized that NAD precursors contained in PPE enhance intracellular NAD production. To test this hypothesis, we analyzed fraction 3 using liquid chromatography–tandem mass spectrometry (LC–MS/MS) to detect the NAD precursors. The results showed that three ion peaks consistent with NMN (*m/z* 335 → 123), NR (*m/z* 255 → 123), and NAM (*m/z* 123 → 80) were detected in fraction 3 (Fig. 3C). The quantification analyses revealed that the concentration of NMN, NR, and NAM contained in whole PPE were 0.22, 17.62, and 0.14 ng/mg, respectively (Table 1). These results suggest that at least three NAD precursors, NMN, NR, and NAM, are present in PPE and participate in intracellular NAD-boosting.

**Table 1:** NAD precursor contents of PPE.

Analytes	Retention time (min)	MRM transitions (*m/z*)		Concentration (ng/1 mg PPE)
		Precursor	Product	
NMN	1.62	335	123	0.22
NR	2.04	255	123	17.62
NAM	4.85	123	80	0.14

### Topical administration of PPE on reconstructed human epidermis increases NAD levels of tissues

**Figure 4:**
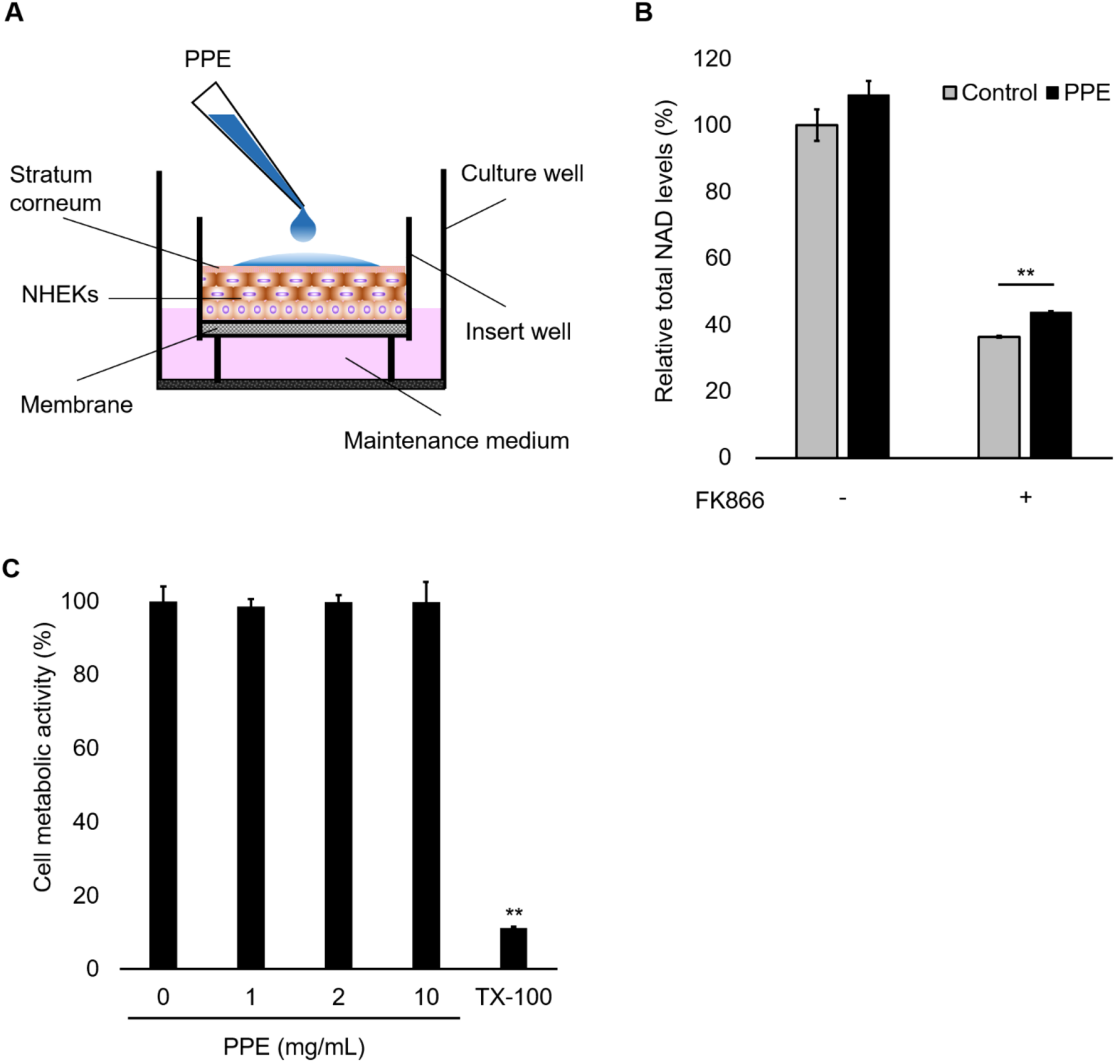
Topical PPE shows NAD recovery activity, but does not affect cell metabolic activity, in RHE. (**A**) Schematic illustration of the RHE model. (**B**) PPE (2 mg/mL) was applied to the stratum corneum side of RHE and incubated for 48 h in the presence or absence of FK866 (5 nM). The total NAD levels of tissues were determined using NAD^+^/NADH assay kit. (**C**) PPE (0, 1, 2, or 10 mg/mL) or 1% TX-100 (as the positive control) was applied to the stratum corneum side of RHE and incubated for 48 h. The cell metabolic activity was determined by MTT assay. **p < 0.01, the comparison to the control.

The primary active ingredients in the NAD-boosting effect of PPE are small molecules, which may be expected to have higher transdermal absorbability than larger proteins such as growth factors. Therefore, topical application of PPE to the skin appears to be a suitable option for increasing NAD levels in skin tissues. To investigate whether topical administration of PPE affects NAD levels in the epidermis below the stratum corneum, 3-dimensional reconstructed human epidermis (RHE) was treated with PPE on the stratum corneum side and cultured in a medium with the presence or absence of FK866 (Fig. 4A). Topical application of PPE increased NAD levels by 9.1% (not significant) or 7.3% (significant) in the absence or presence of FK866, respectively (Fig. 4B). PPE had no effect on cell metabolic activity when tested up to 10 mg/mL, whereas 1% TX-100 markedly reduced (Fig. 4C). These results suggest that the active ingredients in PPE may infiltrate the subcorneal skin and increase skin NAD levels without causing harmful effects.

## Discussion

Many studies have shown that the decrease in NAD due to aging is correlated with the functional decline of tissues and the development of age-related diseases and cancer^11,22^. In the epidermis, UV irradiation, the most important extrinsic factor of skin aging, causes NAD depletion as well as chronological aging^23^. Since the skin is constantly exposed to UV irradiation in daily life, the topical application of NAD-boosting agents seems to be a reasonable approach for preventing NAD reduction and protecting the skin from UV damage. Indeed, enhancing NAD in the skin has been shown to prevent UV-induced skin cancer and immunosuppression in mice^24^. Jacobson et al. reported that topical nicotinic acid derivatives increased skin NAD content and improved barrier function^25^. In addition, several clinical studies have shown that topical application of the NAD precursor NAM reduces signs of skin aging^26,27^. These in vivo and clinical data support the possibility that elevated NAD levels prevent skin damage and delay aging. We have also previously reported that maintaining adequate NAD levels is essential for the protection of human epidermal cells from UV stress^21^. In this study, we demonstrated that PPE enhances intracellular NAD levels in keratinocytes. We also found that its active ingredients have LMW of less than 3 kDa, which are effective for both monolayer and 3-dimensional epidermis culture models. These findings suggest that the active ingredients of PPE could be transdermally absorbed and therefore highlight its potential application as a topical agent for boosting skin NAD levels.

In the skin, NAD deficiency lowers the function of the main energy metabolic pathways, such as glycolysis and oxidative phosphorylation. Glycolysis is indispensable for maintaining a balance between keratinocyte proliferation and differentiation^28^. UV exposure decreases NAD levels and therefore may accelerate this imbalance. The ratio of differentiation to proliferation was observed to be profoundly increased in sun-exposed skin compared to sun-protected skin^29^. Tan et al. found that FK866 treatment accelerated differentiation and decreased metabolic activity in NHEKs, and that NAM reversed these FK866-induced events by restoring cellular NAD levels^28^. In the present study, we found that PPE restored the FK866-induced NAD depletion. Therefore, determining whether PPE can sustain differentiation and proliferation in the NAD-depleted epidermis may provide a better understanding of its anti-aging mechanism.

Recently, Aioi et al. showed that topical use of PPE greatly reduced facial wrinkles and improved skin hydration^17^. Our previous clinical test showed that topical application of PPE cream resulted in a significant improvement in anti-skin aging parameters, such as wrinkles, skin hydration, sagging, and eyebags (unpublished data). The findings of this study may contribute to the understanding of the underlying mechanism by which PPE reduces wrinkles and improves hydration of the skin. A previous report showed that intracellular total NAD levels are positively correlated with the expression of extracellular matrix components, such as procollagen and elastin, in human skin fibroblasts^30^. The treatment of fibroblasts with PPE results in increased production of type I collagen^17^. Combined with these facts, PPE may promote collagen production by increasing NAD content within fibroblasts, which may lead to the alleviation of skin wrinkles observed in clinical trials. Skin hydration is closely associated with barrier function of the epidermal layer. Several studies have suggested that NAD is an essential co-factor for maintaining skin barrier function. Ming et al. showed that the NAD-dependent deacetylase SIRT1 regulates the expression of filaggrin, which plays a critical role as a precursor protein of natural moisturizing factors in skin barrier integrity^31^. In addition, a clinical study demonstrated that the topical application of an NAD-boosting agent markedly decreased transepidermal water loss, together with an increase in stratum corneum thickness in photodamaged skin^25^. These reports suggest that PPE may enhance skin barrier function by increasing cellular NAD levels in the epidermis, probably leading to the improved skin hydration noted in clinical trials.

In recent years, NAD boosters have been shown to be useful in enhancing antioxidant and anti-inflammatory effects. In diabetic and septic mouse models, NR administration significantly reduced high-fat diet-induced brain inflammation and prevented lipopolysaccharide-induced endothelial oxidative stress, respectively^32,33^. Similar to NR, NMN supplementation in aged mice has been shown to reverse the pro-inflammatory mRNA expression profile in the neurovascular unit and to improve cardiovascular function by decreasing oxidative stress^34,35^. In addition, a clinical trial in aged men revealed that oral NR decreased the levels of circulating inflammatory cytokines^36^. Taken together, these in vivo and clinical studies support the idea that the application of NAD-boosting agents provides antioxidant and anti-inflammatory effects. Enhanced SIRT activity has been suggested as one of the plausible mechanisms by which these NAD boosters exert anti-inflammatory and antioxidant effects. PPE is known to have antioxidant and anti-inflammatory effects^15^; however, relatively limited mechanistic exploration has been conducted to date. The present study provides an at least a partial explanation of the effects of PPE.

PPE is known to contain abundant cell growth-promoting proteins such as laminin, cytokines, and growth factors. Recent studies, including ours, have implicated cellular NAD levels to be associated with cell growth in epidermal keratinocytes^21,28^. Hence, we speculated that some of these proteins might contribute to the increase in NAD content. However, contrary to our expectations, we found that a portion containing low-molecular-weight molecules of less than 3 kDa promoted NAD production in keratinocytes. Interestingly, fractionation of the PPE portion using HPLC revealed that NAD production was only observed in fraction 3. We also found that precursors of NAD, such as NMN, NR, and NAM, were present in this fraction, suggesting that they could explain the effect of PPE on NAD production, at least in part. However, since each constituent contained in PPE was detected at very low concentrations, it seems difficult to explain that these constituents alone are responsible for the ability of PPE to promote NAD content in keratinocytes. Recent reports have shown that the reduced forms of these NAD precursors, NRH and NMNH, have a greater ability to produce NAD than NR and NMN^37–40^. Although further analyses are needed to determine whether PPE contains NRH and NMNH, the NAD-boosting ability of PPE observed in this study may be attributed to multiple NAD enhancers, including NMN, NR, NMNH, NRH, and other unknown NAD precursors.

In conclusion, our findings open the possibility of using PPE to improve various diseases and symptoms by increasing the intracellular levels of NAD, an essential co-factor involved in various cellular biochemical reactions.

## Materials and methods

### Preparation of PPE

PE was isolated from frozen porcine placenta. After removing all traces of blood and umbilical cord, the porcine placenta was washed, homogenized, and subjected to three freeze–thaw cycles. The tissue homogenate was centrifuged at 120×*g* for 1 min and the supernatant was collected and used as PPE. To calculate the dry solid content, the PPE was concentrated and dried at 105 °C for 5 h. After cooling to room temperature in a desiccator, the weight was measured with a precision balance. The dry solid content of PPE was 10.98 mg/mL.

### Cell cultures

NHEKs (newborn/male, Thermo Fisher Scientific, Waltham, MA, USA) were cultured in EpiLife™ medium containing 60 μM calcium supplemented with human keratinocyte growth supplement (both from Thermo Fisher Scientific) at 37 °C in a humidified atmosphere with 5% CO_2_. The medium was changed every alternate day until the culture reached approximately 50% confluence, after which it was changed every day. The cells were passaged when they reached 80–90% confluency. NHEKs were not used beyond passage 5.

### NAD quantification and recovery assay

The amount of NAD was measured using the NAD^+^/NADH Assay Kit-WST (Dojindo Laboratories, Kumamoto, Japan) according to the manufacturer’s instructions. Briefly, 1.0 × 10^5^ NHEKs were seeded into 12-well plates. After 24 h, cells were treated with PPE at different concentrations (0, 0.25, 0.5, and 1 mg/mL) and cultured for 1–24 h in the presence or absence of 5 nM FK866 (AdooQ Bioscience, Irvine, CA, USA). The cells were lysed with the supplied NAD^+^/NADH extraction buffer and ultra-filtered using an Ultracel-10 K centrifugal filter device (Merck, Darmstadt, Germany). To calculate NADH concentration, half of the filtrates were incubated at 60 °C for 60 min to decompose NAD^+^. All filtrates were subjected to the enzymatic reaction at 37 °C for 1 h. Then, the absorbance of the samples was measured at 450 nm using an Infinite 200 Pro plate reader (Tecan, Männedorf, Switzerland). The NAD^+^ content was calculated by subtracting the NADH content from the total NAD content. NAD^+^/NADH levels were normalized to the respective protein concentrations determined using the Pierce BCA protein assay (Thermo Fisher Scientific).

### MTT assay

The NHEKs were seeded in a 24 well plate at 0.5 × 10^5^ cells per well and incubated for 24 h. The cells were then treatment with PPE (0.5, 1, 2, and 5 mg/mL) or 0.2% TX-100 for 24 h. The cells were incubated with 300 μL of 1 mg/mL MTT (Thermo Fisher Scientific) solution at 37 °C and 5% CO_2_ for 3 h. After washing three times with PBS, formazan infiltrated in NHEKs was extracted with 200 μL of dimethyl sulfoxide at room temperature for 2 h. Optical absorption at 560 nm was determined using an Infinite 200 Pro plate reader (Tecan).

### Ultrafiltration of PPE

PPE (500 μL) was applied to an Ultracel-3K centrifugal filter device (Merck Millipore) and centrifuged at 16,100×*g* at 4 °C for 60 min. The filtrates were used as a LMW fraction of PPE. To recover the residues on the filter, the filter devices were placed upside down in a new centrifuge tube and centrifuged at 1000×*g* at 4 °C for 5 min. The residues were reconstituted with 500 μL of phosphate-buffered saline (PBS) and used as a HMW fraction of PPE.

### HPLC fractionation

For fractionation of LMW-PPE, we employed an HPLC gradient system equipped with a syringe-loading sample injector, dual pumps, an MD-2010 multi-UV detector (Jasco, Tokyo, Japan) and a C 18 reversed-phase column (Inertsil ODS-3, 5 μm, 4.6 × 250 mm, GL Science, Tokyo, Japan). For high reproducibility, an analytical column was used for fractionation rather than a preparative one. The HPLC solvents were 0.05% trifluoroacetic acid in water (A) and HPLC-grade acetonitrile (B). One hundred microliters of the LMW-PPE was applied to the HPLC column and separated using the following gradient at a flow rate of 1 mL/min: hold at 100% solvent A from 0 to 10 min, then linear gradient from 0% B in 10 min to 95% B in 30 min. The eluents were collected in sample tubes every 2 min and dried using a centrifugal evaporator CVE-3100 (EYELA, Tokyo, Japan). The residues were reconstituted with 100 μL of PBS and filtered through a 0.22 μm membrane before using the NAD recovery assay.

### Analyses of NAD precursors

NMN, NR, and NAM were detected and quantified by isotope dilution LC–MS/MS. Analyses were performed using an ACQUITY UPLC H-Class system coupled with a TQS-micro triple quadrupole mass spectrometer (Waters, Milford, MA, USA) with electrospray ionization operated in positive ion mode. A Capcell Pak ADME HR column (2 μm, 2.1 × 100 mm, Osaka Soda, Osaka, Japan) was used to separate each sample. Mobile phases A–B were water–methanol (containing 0.1% formic acid and 10 mM ammonium formate). The gradient condition was as follows: 0–0.5 min (99% A), 0.5–3 min (99–60% A), 3–5 min (60–2% A), and 5–7 min (2% A). The flow rate was 0.2 mL/min. The capillary voltage was 0.75 kV, source temperature was 150 °C, and desolvation temperature was 500 °C. The cone gas flow was 50 L/h and the desolvation gas flow was 1100 L/h. To quantify each NAD precursor, NAM-^13^C_6_ was added to the samples at a concentration of 78 nM as an internal standard. The ions were monitored using multiple reaction monitoring (MRM) with the following transitions: *m/z* 335 → 123 for NMN, *m/z* 255 → 123 for NR, *m/z* 123 → 80 for NAM, and *m/z* 129 → 85 for NAM-^13^C_6_. NMN was purchased from the Oriental Yeast (Tokyo, Japan). NR and NAM-^13^C_6_ were obtained from Merck (Darmstadt, Germany). NAM was purchased from Wako Pure Chemical Industries (Osaka, Japan).

### RHE

The in vitro RHE model EpiDerm™ (EPI-200) was purchased from MatTek (Ashland, MA, USA) and cultured in EPI-100-ASY medium (MatTek) at 37 °C and 5% CO_2_. One hundred microliters of PPE (1, 2, and 10 mg/mL) or 1% TX-100 was applied to the stratum corneum side of the RHE. The RHE was incubated for 48 h in EPI-100-ASY medium in the presence or absence of FK866 (5 nM). After incubation, the RHE was washed three times with PBS. For the NAD recovery assay, the total NAD levels in RHE were quantified using the NAD^+^/NADH Assay Kit-WST. For the cell metabolic activity assay, the RHE was transferred to a 24-well plate and incubated with 300 μL of 1 mg/mL MTT (Thermo Fisher Scientific) solution at 37 °C and 5% CO_2_ for 3 h. After washing three times with PBS, formazan infiltrated in RHE was extracted with 2 mL of dimethyl sulfoxide at room temperature for 2 h. Optical absorption at 560 nm was determined using an Infinite 200 Pro plate reader (Tecan). The absorbance with 1% TX-100 treated RHE was used as the positive control for cell metabolic activity assay.

### Statistical analysis

Data are presented as the mean ± standard error of the mean of at least three independent experiments. All statistical analyses were performed using the EZR software version 1.55 (Saitama Medical Center, Jichi Medical University, Saitama, Japan). Prior to data comparison, the normality of distribution was confirmed by the Shapiro–Wilk test. The homogeneity of variances was assessed with the F test (two groups) and Bartlett test (multiple groups). The differences among multiple groups were determined using one-way ANOVA followed by Tukey’s post hoc test. Student’s t-test was used to test for significant differences between the two groups. Statistical significance was set at *p* < 0.05 and *p* < 0.01. Asterisks and daggers indicate statistical significance compared to the control (*p < 0.05, **p < 0.01) and indicated (^†^*p* < 0.05 and ^††^*p* < 0.01) groups, respectively.

### Ethical approval

Not applicable. The porcine placentas that were taken and immediately frozen within 3 h after spontaneous delivering of the fetuses were purchased from a farm in Hokkaido, Japan, with the consent of the owner. No live animal has been involved in this study.

## Data Availability

The datasets generated during and/or analyzed during the current study are available from the corresponding author on request.

## Abbreviations

HPLC: High performance liquid chromatography
LC–MS/MS: Liquid chromatography–tandem mass spectrometry
MTT: 3-(4,5-Dimethylthiazol-2-yl)-2,5-diphenyltetrazolium bromide
NAD: Nicotinamide adenine dinucleotide
NADH: Reduced NAD
NAM: Nicotinamide
NAMPT: Nicotinamide phosphoribosyltransferase
NHEKs: Normal human epidermal keratinocytes
NMN: Nicotinamide mononucleotide
NMNH: Reduced NMN
NR: Nicotinamide riboside
NRH: Reduced NR
PARP: Poly ADP-ribose polymerase
PBS: Phosphate-buffered saline
PE: Placental extract
PPE: Porcine PE
HPE: Human PE
HMW-PPE: High molecular weight fraction of PPE
LMW-PPE: Low molecular weight fraction of PPE
RHE: Reconstructed human epidermis
SIRT: Sirtuin
TX-100: Triton X-100
